# Tunable geometrical frustration in magnonic vortex crystals

**DOI:** 10.1038/s41598-017-17480-1

**Published:** 2018-01-09

**Authors:** Carolin Behncke, Christian F. Adolff, Sebastian Wintz, Max Hänze, Benedikt Schulte, Markus Weigand, Simone Finizio, Jörg Raabe, Guido Meier

**Affiliations:** 1 Institut für Angewandte Physik und Zentrum für Mikrostrukturforschung, Universität Hamburg, Jungiusstr. 11, 20355 Hamburg, Germany; 2 The Hamburg Centre for Ultrafast Imaging, Luruper Chaussee 149, 22761 Hamburg, Germany; 3 Paul Scherrer Institut, 5232 Villigen PSI, Switzerland; 4 Max-Planck Institute for Solid State Research, Heisenbergstr. 1, 70569 Stuttgart, Germany; 5 Max-Planck Institute for the Structure and Dynamics of Matter, Luruper Chaussee 149, 22761 Hamburg, Germany; 6 Max-Planck Institute for Intelligent Systems, Heisenbergstr. 3, 70569 Stuttgart, Germany

## Abstract

A novel approach to investigate geometrical frustration is introduced using two-dimensional magnonic vortex crystals. The frustration of the crystal can be manipulated and turned on and off dynamically on the timescale of milliseconds. The vortices are studied using scanning transmission x-ray microscopy and ferromagnetic resonance spectroscopy. They are arranged analogous to the nanomagnets in artificial spin-ice systems. The polarization state of the vortices is tuned in a way that geometrical frustration arises. We demonstrate that frustrated polarization states and non-frustrated states can be tuned to the crystal by changing the frequency of the state formation process.

## Introduction

Frustration is of great general interest as it gives rise to new physical phenomena, such as magnetic monopoles^1,2^. Frustration arises when competing interactions cannot be all satisfied at the same time. The geometrical frustration in well-ordered structures has been studied intensively, e.g. in natural and artificial spin ice systems^3–8^. In natural spin ice system the behaviour of single spins is inaccessible, a drawback that has been overcome by artificial spin ice systems^9^. We combine this research field with magnetic vortices that can be described as magnonic crystals when arranged periodically^10–13^. Geometrical frustration is observed in vortex crystals that are positioned analogous to the nanoislands in artificial spin ice. The behaviour of each vortex in the crystal is directly observable. Additionally, the crystal properties of magnonic vortex crystals can be manipulated dynamically on the timescale of milliseconds^14,15^. In contrast to artificial spin ice systems, the frustration in the vortex crystals can thus be tuned and turned on and off at will. In the non-frustrated state, effects that are linked to the crystal itself, e.g., crystal defects become visible. If the frustration is then turned on, the pure impact of the frustration is observable by comparison with the non-frustrated state. Our study thus paves the way for a dynamic investigation of geometrical frustration itself.

The magnetic vortex state forms in ferromagnetic nanodisks of suitable geometry and features an in-plane curling magnetization with an out-of-plane component in the centre region, the vortex core^10^. The vortex is described by two state parameters: the polarization of the core pointing either up or down (*p* = ± 1), and the circularity, the sense of the in-plane magnetization curling either clockwise or counter-clockwise (*c* = ± 1)^11^. The vortex core can be resonantly excited by magnetic fields and electric currents^16,17^. This leads to a gyrotropic motion of the vortex around its centre position with a resonance frequency in the sub-gigahertz regime^18,19^. The sense of gyration depends on the polarization^16^. Excited vortices can couple via their rotating magnetic stray fields^20^. The collective motions of an arrangement of vortices can be understood by the oscillations of dipolar coupled vortices as building blocks. Periodic arrangements of vortices can be described as magnonic crystals that feature common concepts of solid state physics, e.g. a group velocity, a density of states, and a band structure^14,15,21^. The circularity can be manipulated using static external magnetic fields^22,23^ but the influence on the coupling strength is negligible for dynamically excited two-dimensional arrangements. The coupling strength of neighbouring gyrating vortices in a crystal depends on their relative distance *d*_rel_ and their relative polarization orientation^24^. The coupling can be neglected for relative distances ${d}_{{\rm{rel}}}=\frac{D}{2r} > 2$, with the centre-to-centre distance *D* and the disk radius *r* ^25,26^. The polarization state and thereby the crystal properties can be tuned self-organized using a high frequency magnetic field^15,27^.

**Figure 1:**
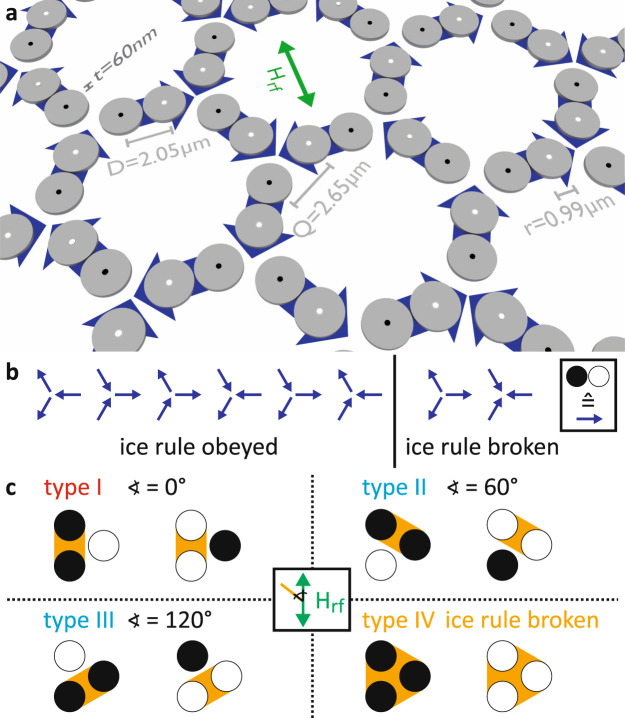
Illustration of the emergent ice rule in magnonic vortex crystals. (**a**) Schematic representation of the investigated vortex system. The black and white dots represent the polarization of the vortices. The blue arrows illustrate the analogy of two vortices with alternating polarizations to a nanoisland in artificial spin ice systems. (**b**) Possible configurations of six vortices in a junction depicted by blue arrows. Configurations that obey the ice rule have two arrows pointing towards the junction and one pointing out of it or vice versa. If all three arrows point in or out of the junction the ice rule is broken. (**c**) Possible polarization configurations of the vortices of a triple, where white and black dots represent a polarization of *p* = +1 and *p* = −1, respectively. The exciting magnetic field breaks the symmetry. It is distinguished between four different triple types. For triple type I-III the ice rule is obeyed, for triple type IV the ice rule is broken.

In this work, we study two-dimensional magnonic vortex crystals that are arranged analogous to the nanoislands in artificial kagome spin-ice (see Fig. 1a). We use ferromagnetic resonance spectroscopy (FMR) to observe the collective behaviour of the system in the frequency domain. Scanning transmission x-ray microscopy (STXM) measurements at the MAXYMUS microscope of the BESSY II synchrotron in Berlin, Germany and at the PolLux endstation at the SLS in Villigen, Switzerland are used to observe the vortex core motions temporally and spatially resolved. We show that it is possible to tune the polarizations of a vortex crystal in a way that frustration during the self-organized state formation arises. Additionally, non-frustrated polarization states can be tuned. The frustration of the system can thus be turned on and off dynamically.

Artificial spin ice systems consist of nanoislands that are in a single domain state and are described as Ising spins^5^. The alignment of these spins follows the ice rule^28^. In the hexagonal kagome spin ice, a junction consists of three nanoislands. The preferred state is reached if all spins do not point towards or away from the junction. Since the overall spin has always a finite value, the system is frustrated^29^. In our system, two vortices with alternating polarization are analogous to one nanoisland. Thus, the hexagons consist of twelve vortices. The vortices in the pairs are in close proximity and are strongly coupled (${d}_{{\rm{rel}}}=\frac{D}{2r}=1.035$, see Fig. 1a). Vortices in triple junctions at the connections of the hexagons are weaker coupled (${d}_{{\rm{rel}}}=\frac{{Q}}{2r}=1.338$). If each vortex in the triple junction prefers an alternating polarization with each neighbour, frustration arises. We will show that this situation can be created by an appropriate choice of the polarization-state formation process. For the comparison with a nanoisland in artificial spin ice, a vortex pair with alternating polarization is represented by a blue arrow pointing from a polarization of *p* = −1 (black dots in Fig. 1) to the vortex with a polarization of *p* = +1 (white dots in Fig. 1). The eight possible configurations of arrows in a junction are shown in Fig. 1b. If the ice rule is obeyed, two arrows point into the centre and one points out of it, or vice-versa. If all three arrows point in or out of the centre, the ice rule is broken^6^. For the tuning of the polarization configuration, the vortex crystal is excited by a uniaxial high frequency magnetic field. Because of the different alignment of the polarization state with the field, the symmetry is broken. The six vertex types that obey the ice rule (Fig. 1b) cannot be considered equally. We distinguish four different types of junctions that are illustrated in Fig. 1c. For clarity only the three vortices in the centre are shown. For triples of type I the two vortices of equal polarization (marked in orange in Fig. 1c) are aligned parallel to the exciting magnetic field. For type II the angle is 60° and for type III 120°. Triples of type IV have equal polarizations and the ice rule is broken.

## Results

### Samples and state formation

**Figure 2:**
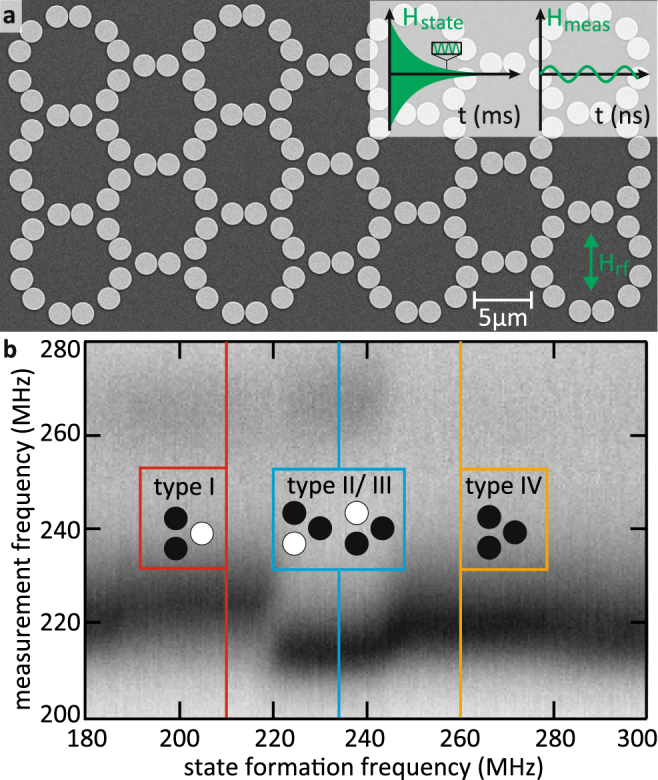
Scanning electron micrograph of the sample and FMR measurements. (**a**) Scanning electron micrograph of the investigated sample. A copper stripline (not shown) is placed upon the permalloy disks to excite the gyrational motion of the vortices. The polarization pattern is tuned via an adiabatic reduction of a high frequency magnetic field excitation of maximum *μ*_0_ *H*_state_ ≈ 0.7 mT. A low-amplitude harmonic field *μ*_0_ *H*_meas_ ≈ 0.1 mT excites the vortices during the FMR and STXM measurements. (**b**) Ferromagnetic absorption spectra for varying state formation frequencies. The insets show the most common triple types for a state formation frequency of 210 MHz (red), 234 MHz (blue), and 261 MHz (orange). The resonance frequency of an isolated vortex is 247 MHz.

The investigated vortex crystals consist of 146 permalloy (Ni_80_Fe_20_) disks (see Fig. 2a). The disks have a radius of *r* = 990 nm and a height of *t* = 60 nm. The minimal distance between the vortex pairs is 70 nm, whereas the distance between the disks in the weaker coupled triple junctions is 670 nm. A copper stripline (not shown) with a width of 30 μm and a height of 100 nm with a gold capping of 5 nm is placed horizontally upon all the disks for the excitation of the vortex dynamics. A high frequency current sent through the stripline leads to a high frequency Oersted field in the disks’ plane. The insets in Fig. 2a show the two magnetic field signals that are used during the experiments. An adiabatically decreasing high frequency magnetic field is used to tune the polarization configuration in the crystal. At the beginning, the large amplitude of the magnetic field of *μ*_0_ *H*_state_ ≈ 0.7 mT leads to large velocities of the vortices and the vortex cores switch their polarization repeatedly^16,30^. As the amplitude of the magnetic field is reduced on the time scale of milliseconds, the switching dies out eventually. The vortices form the ground-state polarization-configuration that is most stable at the frequency of the exciting field. Depending on the frequency of the state formation signal, different ground states can be tuned. For a detailed explanation of this self-organized state formation process see refs.^27,31^. Here, we compare the polarization state formation to the temperature-induced switching of the magnetization of nanoislands in artificial spin ice systems. In this context, a high magnetic field amplitude *H*_state_ can be understood as a high temperature that activates the vortex core switching. As the field amplitude is reduced, the vortex core switching is frozen out. A low-amplitude harmonic signal of *μ*_0_ *H*_meas_ ≈ 0.1 mT excites the gyrational motion of the vortices without changing the polarizations. It is used for the characterization of the crystal in the spatial and frequency domain. As the polarization state is stable during the measurements, an undisturbed investigation of the system is ensured.

### Pre-characterization with FMR

In a first step, the vortex crystal is pre-characterized using FMR. The polarization state is tuned using the state formation signal *H*_state_. The vortex crystal is then excited by the measurement field *H*_meas_ with different frequencies. This field does not affect the polarization configuration because of its low amplitude. The absorption is determined with the help of a reference measurement at 60 mT in the absence of vortices in the disks. At the strongest absorption the vortices are excited resonantly. Figure 2b shows absorption spectra of the FMR-measurements at different state formation frequencies where black contrast corresponds to strong absorption. Different polarization patterns have different resonance frequencies^32^. For low and high state formation frequencies the measured absorption spectra are identical. They correspond to random polarization states. At intermediate state formation frequencies of 190 − 280 MHz the resonance frequency is shifted. This shift of the resonance frequency is attributed to the formation of ordered polarization states. We observe three regimes of different resonance frequencies. STXM measurements, that are performed in a next step, reveal the corresponding polarization patterns. In these measurements, the vortices are excited non-invasively with a harmonic magnetic field *H*_meas_. For the determination of the vortex polarizations, the gyration of each vortex core of the crystal is recorded. It is observed, that for the three regimes of state formation frequencies different triple types (see Fig. 1c) are preferably tuned to the crystal. The most common triple types of the three state formation frequency regimes are depicted in the insets of Fig. 2b. For a state formation frequency of 210 MHz (red) triples of type I are favoured. For a state formation frequency of 234 MHz (blue) triples of type II and III occur most frequently and for a state formation frequency of 261 MHz (orange) triples of type IV are the most common.

### Frustrated and non-frustrated polarization states

**Figure 3:**
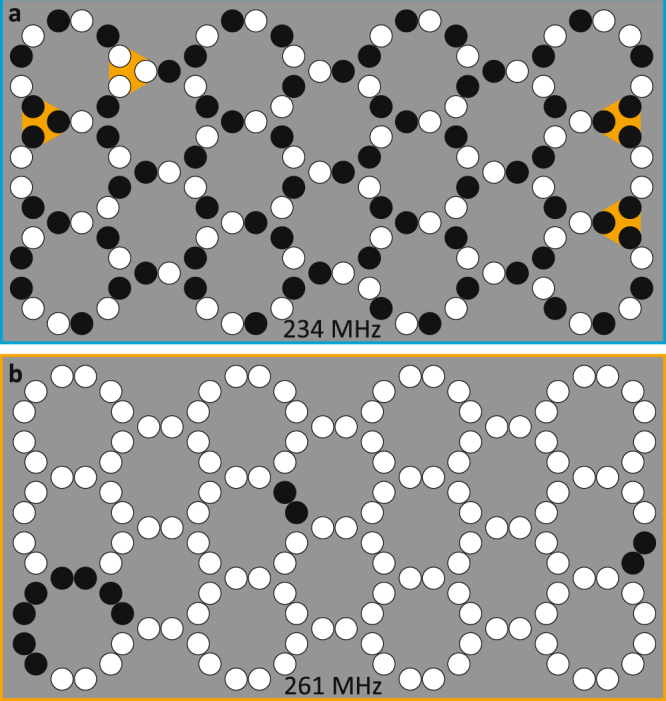
Frustrated and non-frustrated polarization states obtained by STXM measurements. Polarization patterns of the vortex crystal after state formation with a frequency of (**a**) 234 MHz and (**b**) 261 MHz. In (**a**) an alternating polarization of neighbouring vortices is favoured. Exceptions of the ice rules are highlighted in orange. In (**b**) homogeneous polarizations of neighbouring vortices are favoured.

In Fig. 3 the polarization patterns determined by the STXM measurements are shown for state formation frequencies of 234 MHz and 261 MHz for the whole vortex crystal (see also the supplementary material, supplementary movie S1 and S2 for the STXM raw data). For a state formation frequency of 234 MHz (Fig. 3a) the strongly coupled vortex pairs always have alternating polarizations. At this frequency the crystal therefore fulfils the basic requirement to be a frustrated system. In this situation, a vortex pair can be compared to a nanoisland of artificial spin ice systems with a certain alignment of the magnetization. Vortices in triple junctions mostly have the polarization configurations of type II and III. Violations of the ice rule (triples of type IV) are suppressed and occur at the edges of the crystal only. The ice rule violations are marked in orange in Fig. 3a. Due to the symmetry breaking of the exciting magnetic field, triples of type I are also rare. As in spin ice systems there is no long-range order perceptible. That means that even though a highly symmetric state (e.g. consisting of type II triples only) might be the lowest-energy state, it is not necessarily adjusted to the crystal. The data suggest that the vortices favour an alternating polarization configuration with each of their neighbours during the state formation with a frequency of 234 MHz. As this is not possible for three vortices in a triple junction, the polarization state formation process is frustrated.

It is also possible to tune a non-frustrated polarization state to the system. For a state formation frequency of 261 MHz (Fig. 3b) an almost complete homogeneous polarization configuration is tuned. The strongly coupled vortex pairs always have equal polarizations. There are few defects that occur at the weaker coupled triple junctions. Compared to the frustrated state a long-range order is observed.

### Quantitative analysis of occurring triple types

**Figure 4:**
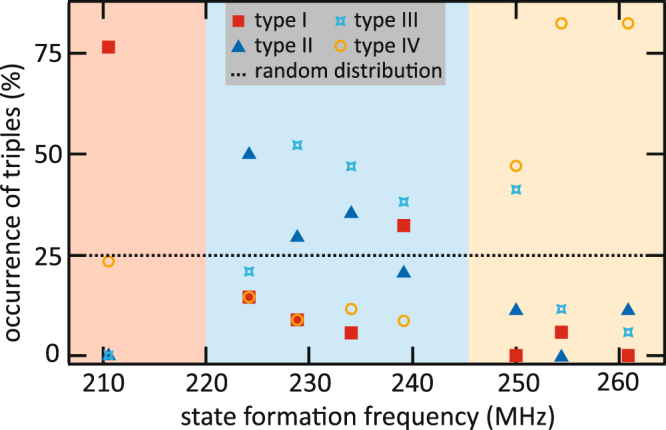
Statistics of the different triple types. Occurrence of the four triple types for different frequencies of the state formation signal. A random distribution would lead to an occurrence of 25% for each triple type (dashed line). The coloured regions refer to the preferred triple type in each region (red: type I, blue: type II & III and orange: type IV) in accordance with the FMR measurements.

For a quantitative description of the polarization states, the occurrence of the four triple types is plotted in dependence of the state formation frequency (Fig. 4). As mentioned in the description of Fig. 2b, there are preferred triple types depending on the state formation frequency. The data points are obtained by analysing the polarizations of each vortex in the crystal after polarization state formation with different frequencies. The corresponding polarization configurations are depicted in the supplementary material. If the polarizations were randomly distributed, each triple type would occur with a probability of 25% (dashed line in Fig. 4). The coloured backgrounds correspond to the preferred triple types in the sections, i.e. the frequency regimes in the FMR measurements. For low state formation frequencies triples of type I occur most frequently. For intermediate state formation frequencies triple types II or III are the most common, whereas triple type I and IV are mostly suppressed. Triples of type IV are favoured for high state formation frequencies, for which the other triple types rarely occur. The pairs of strongly coupled vortices favour an alternating polarization configuration in the blue area regardless of their orientation. Here, the system is frustrated. Homogeneous polarization pairs are favoured in the orange area where no frustration occurs. For small state formation frequencies (red) the favoured pair configuration depends on the angle between the vortex pair and the exciting magnetic field. The plot shows that the polarization state can be tuned in a way that a favoured triple type is dominant. It also shows that ice rule violating triple types are suppressed for the mid-range state formation frequencies. Despite the difference of the symmetry breaking magnetic field, the polarization state strongly resembles a frustrated artificial spin ice state. For a state formation frequency of 239 MHz triple types I, II, III are nearly equally distributed, which means that here these three triple types are equally stable. Thus, the tuning of the polarization pattern not only enables us to turn the frustration on and off but also to manipulate the frustration itself.

## Conclusion

We have shown that geometrical frustration occurs in magnonic vortex crystals during the process of polarization state formation. This frustration only occurs in a certain frequency range of the state formation signal. For other frequencies the system is not frustrated and a long-range polarization order is observed. In the frustrated case, only short range order plays a role. It is possible to change the polarization configuration from a frustrated state to a non-frustrated state and vice-versa. Our study shows that it is possible to tune and to turn the frustration on and off within milliseconds. This allows future studies to compare the frustrated and the non-frustrated state of an otherwise identical system to gain deeper insight into the geometrical frustration itself.

## Methods

### Sample preparation

Vortex crystals consisting of 146 permalloy (Ni_80_Fe_20_) disks are prepared by electron-beam lithography, O_2_ plasma etching, thermal evaporation, and lift-off processing on 100 nm thick silicon nitride membranes transparent for soft x-rays. The radius and height of the disks are *r* = 990 nm and *t* = 60 nm. The minimal distance between the vortex pairs is 70 nm and in the triple junctions the minimal distance is 670 nm. A copper stripline with a width of 30 μm and a height of 100 nm with a gold capping of 5 nm is placed horizontally upon all the disks for the excitation of the vortex dynamics.

### STXM measurements

The trajectories of the vortex core motions of all 146 disks of a vortex crystal are directly observed using STXM at the MAXYMUS microscope of the BESSY II synchrotron in Berlin, Germany and at the PolLux endstation at the SLS in Villigen, Switzerland. The magnetic contrast is provided via the x-ray magnetic circular dicroism at the Ni *L*_3_ absorption edge (*E* ≈ 855 eV). The setups provide a spatial resolution of about 25 nm and a temporal resolution of about 70 ps. The dynamics of the out-of-plane component of the magnetization, i.e. the vortex cores, were stroboscopically imaged with the sample placed perpendicular to the beam axis. The polarization of the vortex cores is determined by the magnetic contrast (white and black dots corresponding to a polarization of *p* = +1 and *p* = −1, respectively). The sense of gyration of the cores (counter-clockwise for *p* = +1 and clockwise for *p* = −1, respectively) provides the equivalent information.

### Data availability

All data generated or analysed during this study are included in this published article (and its Supplementary Information files).

## Electronic supplementary material

Supplementary material

Supplementary Movie S1

Supplementary Movie S2
