# Supplementary material for: Tunable geometrical frustration in magnonic vortex crystals
Source: Sci Rep. 2018 Jan 9;8:186. doi: 10.1038/s41598-017-17480-1 (PMC5760707; doi:10.1038/s41598-017-17480-1)
Supplement: Supplementary file 1 — Supplementary material [file 41598_2017_17480_MOESM1_ESM.pdf]

Tunable geometrical frustration in magnonic vortex crystals

Supplementary material

Carolin Behncke[1,*], Christian F. Adolff[1,2], Sebastian Wintz[3], Max Hänze[4,5], Benedikt Schulte[5], Markus Weigand[6], Simone Finizio[3], Jörg Raabe[3], and Guido Meier[2,5]

[1]Institut für Angewandte Physik und Zentrum für Mikrostrukturforschung, Universität Hamburg, 20355 Hamburg, Germany

[2]The Hamburg Centre for Ultrafast Imaging, Luruper Chaussee 149, 22761 Hamburg, Germany

[3]Paul Scherrer Institut, 5232 Villigen PSI, Switzerland

[4]Max-Planck Institute for Solid State Research, Heisenbergstr. 1, 70569 Stuttgart, Germany

[5]Max-Planck Institute for the Structure and Dynamics of Matter, Luruper Chaussee 149, 22761 Hamburg, Germany

[6]Max-Planck-Institut für Intelligente Systeme, Heisenbergstr. 3, 70569 Stuttgart, Germany

* cbehncke@physnet.uni-hamburg.de

A Movie description

In the movies the pure magnetic contrast of the scanning transmission x-ray measurements is shown. This is achieved by dividing every time frame of the movie by the average frame of the whole movie. The disks have a radius of $r = 0.99\,\mu$m and a thickness of 60 nm. The disk interspace is colorized in coppery depicting the stripline that covers the disks. A radio frequency field H_{meas} created by a current sent through the stripline induces the gyrotropic motion of all vortices. The excitation amplitude is approximately $\mu_0 H_{\mathrm{meas}} = 0.16\,\mathrm{mT}$[1]. The direction of the field is indicated by the green arrow. The vortices appear as white or black dots according to their polarization $p = \pm 1$. White (black) vortex cores gyrate counter-clockwise (clockwise) and thus have a positive (negative) polarization.

Supplementary Movie S1

The movie shows twenty vortices in a cutout of the full crystal that are in a frustrated polarization state. Prior to the measurement the polarization state is tuned using a high frequency magnetic field of 235 MHz that is reduced adiabatically[2]. The maximal amplitude of the state formation signal is $\mu_0 H_{\mathrm{state}} = 0.66\,\mathrm{mT}$. The vortex crystal is captured for one period of excitation. Therefore the measurement field $\mu_0 H_{\mathrm{meas}} = 0.16\,\mathrm{mT}$ with a frequency of 235 MHz is applied. For the convenience of the viewer the data is repeated four times. The time resolution in the movie is 250 ps. The polarization pattern obtained by the movie is depicted in Fig. 1a.

Supplementary Movie S2

The movie shows ten vortices of the crystal that are in a non-frustrated polarization state. The polarization state of the vortices is tuned via a state formation signal with a frequency of 261 MHz. The maximal amplitude of the state formation signal is $\mu_0 H_{\mathrm{state}} = 0.74\,\mathrm{mT}$. The vortex crystal is captured for one period of excitation (261 MHz). For the convenience of the viewer the data is repeated four times. The time resolution in the movie is 167 ps. The polarization pattern obtained by the movie is depicted in Fig. 1b.

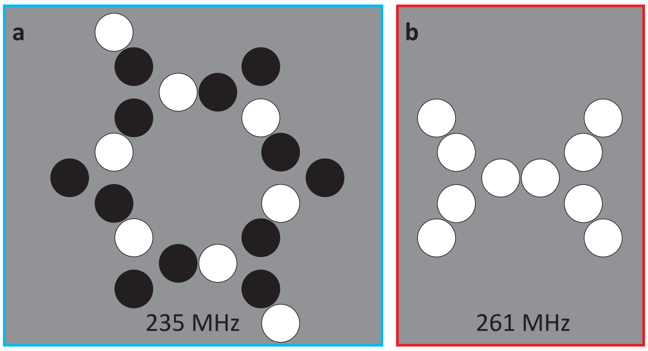

Figure 1 | Polarization patterns obtained from the movies. (**a**) Supplementary Movie S1, 235 MHz, (**b**) Supplementary Movie S2, 261 MHz. White and black dots correspond to a polarization of $p = +1$ and $p = -1$, respectively.

B Polarization states

Figure 2 shows the observed polarization states of the vortex crystal that correspond to the data points of Fig. 4 in the main text. Prior to the measurement a polarization state formation with frequencies between 210 and 261 MHz was performed. The polarization patterns are obtained by scanning transmission x-ray measurements of the magnetic contrast of each vortex core of the crystal. White and black dots depict a polarization of $p = +1$ and $p = -1$, respectively. Grey dots represent non-recorded vortices. In Fig. 2a triples of type I are favored. In Fig. 2b-e an alternating polarization of neighboring vortices is favored. Exceptions of the ice rules are highlighted in orange. In Fig. 2f-h a homogeneous polarization of neighboring vortices is dominant. The colored contours of the images correspond to the favored triple types of the polarization patterns: red: type I, dark blue: type II, light blue: type III, and orange: type IV.

References

1. T.J. Silva, C.S. Lee, T.M. Crawford, and C.T. Rogers. Inductive measurement of ultrafast magnetization dynamics in thin-film Permalloy. *J. Appl. Phys.* **85,** 7849 (1999).

2. C. F. Adolff, M. Hänze, A. Vogel, M. Weigand, M. Martens, and G. Meier. Self-organized state formation in magnonic vortex crystals. *Phys. Rev. B* **88,** 224425 (2013).

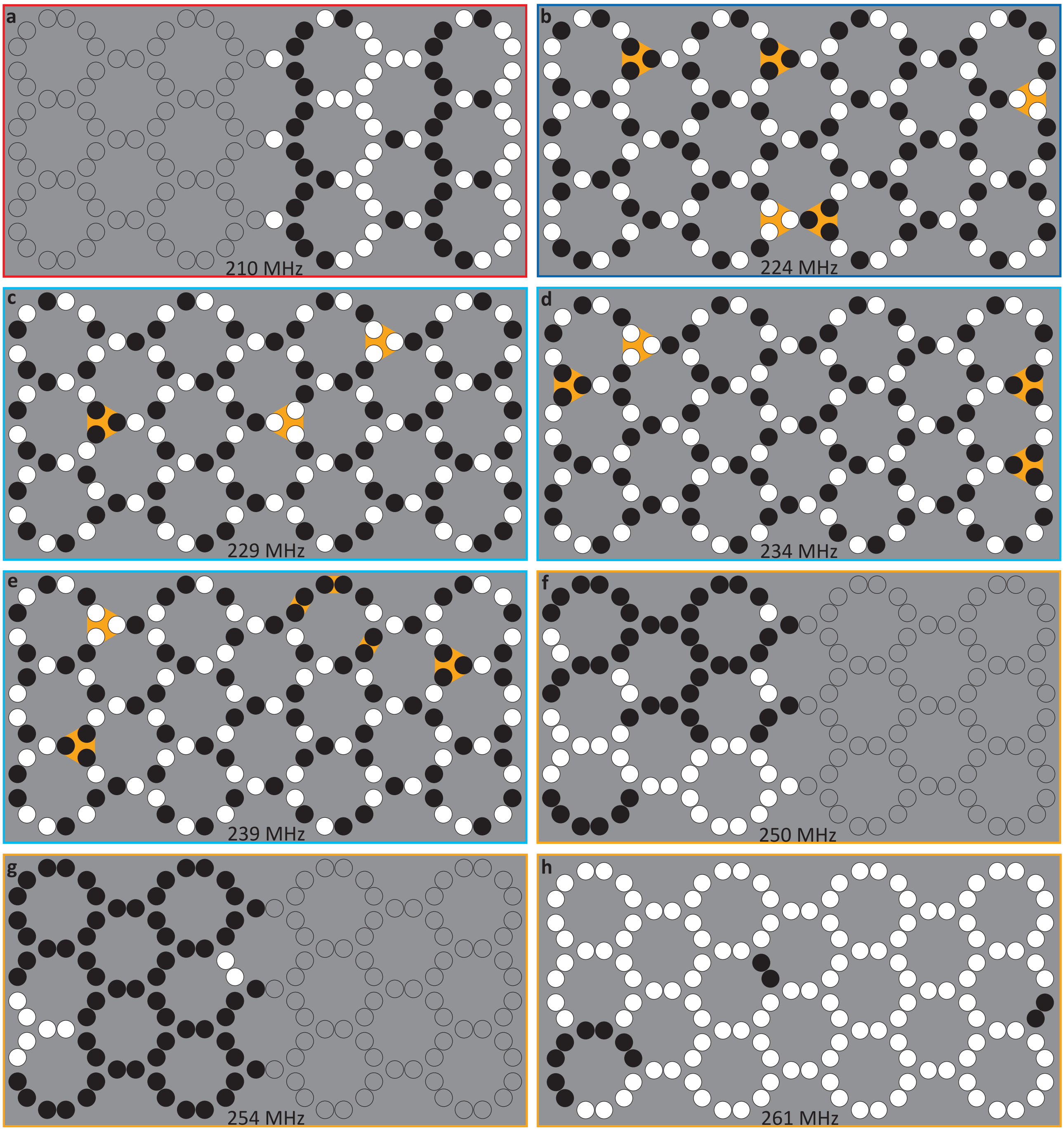

Figure 2 | Polarization patterns of the vortex crystal after state formation with varying frequencies. The following frequencies and maximal amplitudes of the state formation signal are used: (**a**) 210 MHz, 0.66 mT, (**b**) 224 MHz, 0.66 mT, (**c**) 229 MHz, 0.84 mT, (**d**) 234 MHz, 0.66 mT, (**e**) 239 MHz, 0.66 mT, (**f**) 250 MHz, 0.66 mT, (**g**) 254 MHz, 0.66 mT, and (**h**) 261 MHz, 0.74 mT.
